# Complicated Surgical Removal of an Adherent Port-a-Cath After 11 Years of Implantation

**DOI:** 10.7759/cureus.7301

**Published:** 2020-03-17

**Authors:** Divy Mehra, Dieter Brummund, Benjamin Sinyor, Seza Gulec

**Affiliations:** 1 Ophthalmology, Nova Southeastern University Dr. Kiran C. Patel College of Osteopathic Medicine, Miami, USA; 2 General Surgery, Aventura Hospital and Medical Center, Aventura, USA; 3 Surgical Oncology, Herbert Wertheim College of Medicine, Florida International University, Miami, USA

**Keywords:** mediport, nigro, port-a-cath, anal squamous cell carcinoma, superior vena cava, guidewire, central venous catheter, polyurethrane, intravascular adhesion

## Abstract

A 65-year-old woman with a medical history significant for anal cancer was referred by her primary care physician for a port-a-cath removal. The port was placed prior to treatment of squamous cell carcinoma of the anus, 11 years prior to this scheduled removal. She received chemotherapy and radiation in accordance with the Nigro protocol, treating the anal cancer to complete resolution. During port removal, a fibrous capsule was dissected and the port was removed from the left upper breast border along with proximal portion of the catheter. Significant difficulty was found in removing the remaining catheter despite sustained traction and guidewire insertion. Fluoroscopy revealed an intravascular adhesion of the catheter tip in the superior vena cava, which could not be freed. In order to prevent vascular injury, the adhesed portion of the distal catheter was left in place with three large surgical clips placed distally. This case highlights the very rare complication of complete vascular adherence of the terminal catheter tip and extended port intracorporeal time as a risk factor for adhesion. This case also highlights the importance of timely permanent central venous catheter removal following completion of its intended regimen.

## Introduction

A totally implantable venous access device (port-a-cath) is a type of central venous catheter consisting of a surgically placed subcutaneous port attached to a catheter inserted into a major vessel, commonly terminating at the junction of the lower superior vena cava and right atrium. It is designed to allow for repeated parental access for medication, nutrition, and fluid administration as well as a sampling of venous blood [1]. This long-term central venous access is typically used for chemotherapeutic regimens. Removal of the port-a-cath is generally a short, uncomplicated procedure, and should be done soon after completion of the intended regimen. Port removal involves an incision overlying the port reservoir, dissection to the port catheter and reservoir, and gentle traction to remove the catheter, followed by closure of the venous insertion site, tunnel, and wound [2]. While difficulty removing port catheters is uncommon, a long port dwell time (>20 months) has been associated with difficulty in catheter removal even with a strong traction force by adherence to the vessel wall [3]. Few such cases have been documented in the literature, totaling less than 1% of all permanent central venous catheter removal procedures, and adherence of the terminal catheter tip is an even more rare occurrence (1.5%) [3,4]. This case details vascular adhesion at the superior vena cava of the distal catheter tip of a port-a-cath left in place for 11 years, preventing complete removal.

## Case presentation

**Figure 1:**
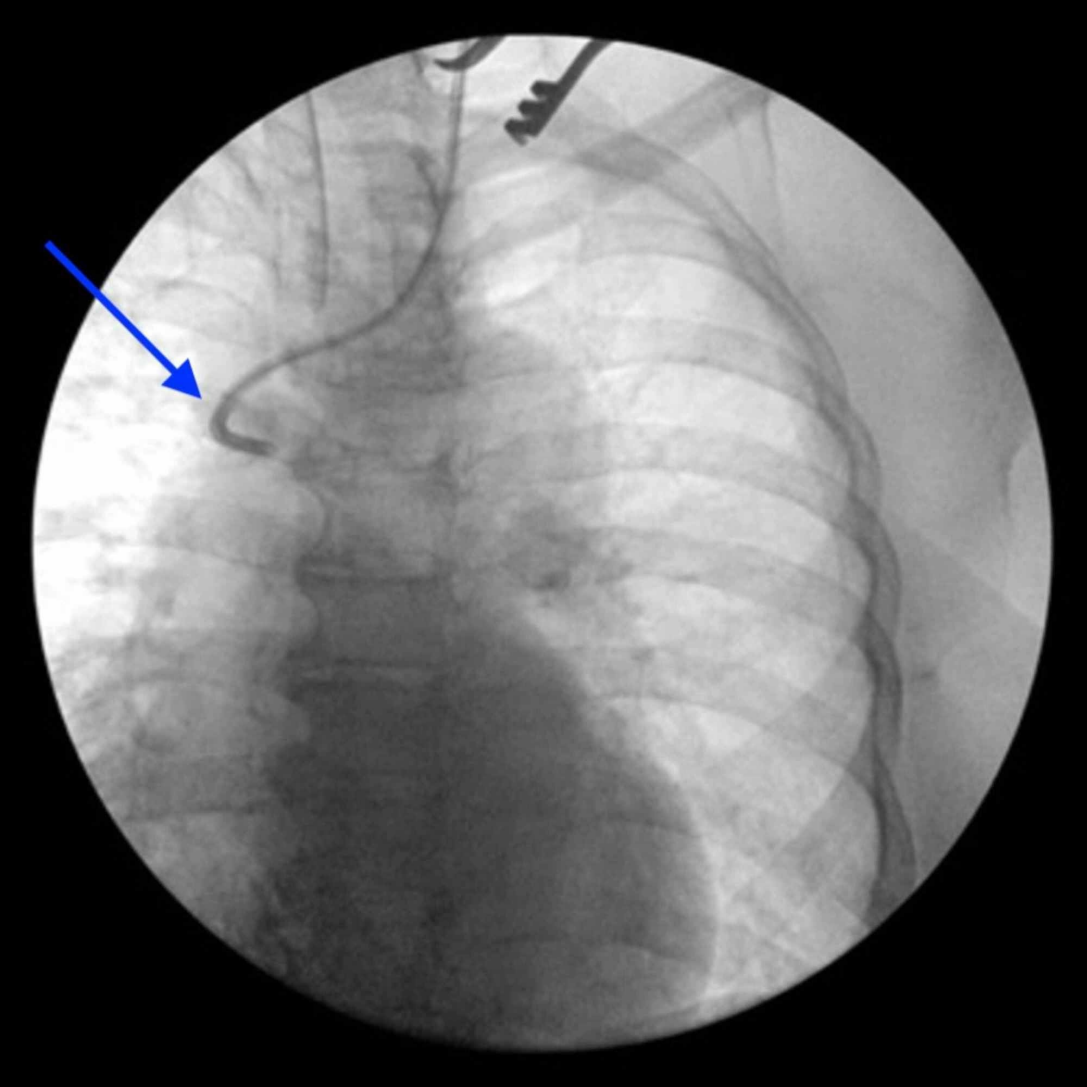
Fluoroscopic examination of distal catheter. The catheter tip was determined to be stuck in superior vena cava.

A 65-year-old woman with a medical history significant for anal cancer presented for mediport removal. Past surgical history included an elective thyroidectomy two years prior for multinodular goiter. The port was placed 11 years prior for chemotherapeutic treatment of anal cancer, which subsequently cleared with complete clinical response following treatment with the Nigro protocol. Thereafter, the patient failed to continue her scheduled postoperative follow-up evaluations. The patient was referred from her primary care physician for port removal, which was proceeded with as requested. The port-a-cath had been inserted into the left internal jugular vein with the catheter tip in the superior vena cava, and the port was secured in the subcutaneous tissue of the left chest wall along the left upper breast border. Under monitored anesthesia care and local anesthesia, an incision was made over the port site, approximately 2 cm inferior to prior incision site. The port and catheter were identified covered with chronic capsule. Subcapsular dissection allowed for complete freeing of the port, but significant resistance was felt upon pulling the catheter. To reduce subcutaneous tunnel resistance along the catheter tract, a second incision was made superior to the primary incision, closer to the site of left internal jugular vein catheter insertion. A clamp was placed on the proximal catheter end and the catheter transected. The explanted metallic port (measuring 2.5 x 2.5 x 1.3 cm) and transected proximal catheter (3.5 cm) were removed. We continued to dissect along the catheter tract to the point of insertion into the left internal jugular vein, followed by venotomy and temporary local tonsil clamp placement. At this point, no reduction in resistance was felt despite sustained traction, suggesting a possible intravascular central vein adhesion to the catheter. A cardiothoracic surgeon was consulted, and the patient was converted from monitored to intratracheal general anesthesia care. A guidewire was inserted followed by no change in resistance of the catheter tip, and there was a confirmed absence of backflow from the catheter. Fluoroscopic imaging was completed (Figure 1), indicating the catheter tip was fixed to the sidewall of the superior vena cava. This fixation made the complete removal of the catheter by traction unsafe. We concluded that further attempt to retrieve the stuck catheter may result in vascular injury, and opted instead to shorten the distal catheter end while leaving the adherent tip in place. Three large surgical clips were placed and the distal catheter was transected (transected piece measuring 11.5 cm in length). All incisions, affected subcutaneous planes, and skin were closed in anatomic planes.

## Discussion

Removal of a totally implantable venous access device is typically a relatively uncomplicated procedure, but cases of difficult removal are documented in the literature as a rare complication of port-a-cath use. A review of port-a-cath removals conducted between 2003 and 2012 identified just 4% as “difficult removals,” of which only 9% were considered intravascular adhesions (n=1,306) [3]. In most patients with intravascular adhesion to “stuck” catheter, dissection of catheter generally allowed resolution of catheter adhesion, except in rare cases of firm adhesion in which catheters were left in situ (1.5%, n=197) [4]. Retention of catheters is considered an adverse event of chemotherapy treatment in regimens consisting of totally implantable venous access device use [4]. Documented risk factors for the development of intravascular adhesion of a port-a-cath include younger age of insertion, increased port dwell time (>20 months), a diagnosis of acute lymphocytic leukemia, and polyurethane catheter use [3,4].

An association with acute lymphoblastic leukemia suggests a vascular or hematologic physiological manifestation of the malignancy or an effect of its chemotherapeutic regimen [3].

The presented case consisted of a port dwell time of 129 months, corroborating prolonged port implantation time as a risk factor for difficult removal. This relationship has been mentioned consistently in reports and the literature; however, given the relatively small sample size of this complication, the precise significance is indeterminate [3]. No specific time frame has been established for intravascular adhesion, and it is not known whether earlier port removal would have corresponded to less adhesion or difficulty in removal.

In a similar case, an alternative approach including a "push-in" force using a guidewire is outlined by Huang et al. [2]. Complications of forceful traction in attempted removal include vascular injury at the site of adhesion and catheter fragmentation, which may be avoided with the use of a guidewire. In the presented case, the insertion of a guidewire provided no decrease in resistance of the catheter, possibly attributed to extensive adhesion or location of resistance localized to the terminal catheter tip. Vascular injury and perforation are complications to be considered during guidewire insertion.

Permanent venous access or hemodialysis catheters may become “stuck” if a fibrin sleeve forms and attaches it to an adjacent venous wall with possible calcification [5]. One technique used to remove incarcerated permanent venous access catheters was introduced by Quaretti et al., as evidenced by successful removal without complications of adhering hemodialysis catheters in four patients - this report was a refinement of two individual techniques introduced by Hong as solutions for a “stuck” catheter [5-7]. In a similar case, a catheter successfully removed after 12 years of implantation revealed a calcified intravascular fibrin sleeve on a computed tomography scan at two-month follow-up [5]. As described by Quaretti et al., the catheter is firstly cut close to its venous entry point, followed by the insertion of a valved introducer for guidewire and balloon access [5]. A stiff guidewire is then inserted into the inferior vena cava to avoid potential complications associated with heart cavity injury. Endoluminal dilations with varying balloon diameters may be monitored with fluoroscopy to localize areas of incarceration and constriction until the catheter is freed and removed. Endoluminal balloon dilatation breaks the adhesions between catheter and adherent vein in addition to expanding the vein lumen, allowing for less resistance to removal [5].

The effects of systemic chemotherapeutic regimens may be considered a precipitating factor in endovascular remodeling or scarring in foreign body reactions. Management of anal squamous cell cancer is guided by the Nigro protocol, first described in 1974. Chemoradiotherapy is preferred over abdominoperitoneal resection, and it remains the gold standard in anal squamous cell cancer treatment due to better local control, lower recurrence, and prolonged survival [8]. National Comprehensive Cancer Network (NCCN) guidelines (2017) for the treatment of locoregional anal squamous cell carcinoma consists of continuous intravenous (IV) infusion 5-fluorouracil days 1-4 and 29-32 (1,000 mg/m^2^/day), IV bolus mitomycin C days 1 and 29 (10 mg/m^2^), and concurrent low-dose intensity-modulated radiation therapy. 5FU is associated with cardiotoxicity, which may manifest as chest pain, acute coronary syndrome/myocardial infarction, arrhythmia, myocarditis and pericarditis, and heart failure. Potential mechanisms of direct cellular or ischemic damage include free radical-induced direct myocardial damage, primary smooth muscle dysfunction, altered red blood cell morphology and oxygen-carrying capacity, and direct endothelial cell damage [9].

Catheters left in situ due to adhesion have not been found to be associated with any complications [3]. A single center study of totally implantable venous access device removal by Wilson et al. revealed no complications with retained catheters in situ without venous thromboembolism prophylaxis at six-year follow-up [4]. Longer-term analysis has not been reported, and potential complications such as local infection and venous thromboembolism cannot be definitively precluded.

## Conclusions

Vascular catheter adhesion is a potential long-term complication of utilizing an implanted venous access device. As a result, we suggest timely removal of port-a-cath following completion of intended chemotherapeutic regimen. In cases of indwelling catheters for extended periods (>20 months), appropriate preparation should be made in anticipating this complication with removal - this may include forewarning the anesthesiologist regarding a potentially longer procedure, ensuring necessary endovascular and imaging equipment is available, and the access to a cardiothoracic surgery or interventional radiology consultation. 

## References

[1] Cerini P, Guzzardi G, Galbiati A, Stanca C, Del Sette B, Carriero A (2017). Endoluminal dilation technique to remove stuck port-a-cath: a case report. Ann Vasc Surg.

[2] Huang S-C, Tsai M-S, Lai H-S (2009). A new technique to remove a “stuck” totally implantable venous access catheter. J Pediatr Surg.

[3] Patel PA, Parra D, Bath R (2016). IR approaches to difficult removals of totally implanted venous access port catheters in children: a single-center experience. J Vasc Interv Radiol.

[4] Wilson GJP, van Noesel MM, Hop WCJ, van de Ven C (2006). The catheter is stuck: complications experienced during removal of a totally implantable venous access device. A single-center study in 200 children. J Pediatr Surg.

[5] Quaretti P, Galli F, Fiorina I (2014). A refinement of Hong’s technique for removal of stuck dialysis catheters: an easy solution to a complex problem. J Vasc Access.

[6] Hong JH (2010). An easy technique for the removal of a hemodialysis catheter stuck in central veins. J Vasc Access.

[7] Hong JH (2011). A breakthrough technique for the removal of a hemodialysis catheter stuck in the central vein: endoluminal balloon dilatation of the stuck catheter. J Vasc Access.

[8] Kaya S, Altin O, Altuntas YE (2019). Anal canal squamous cell cancer: surgıcal therapy, when?. Med Glas (Zenica).

[9] Jaskanwal SD, Kaur J, Khodadadi R (2018). 5-fluorouracil and cardiotoxicity: a review. Ther Adv Med Oncol.

